# Functional rare variant in a *C/EBP* *beta* binding site in *NINJ2* gene increases the risk of coronary artery disease

**DOI:** 10.18632/aging.203755

**Published:** 2021-12-12

**Authors:** Pengyun Wang, Yifan Wang, Huixin Peng, Jingjing Wang, Qian Zheng, Pengxia Wang, Jing Wang, Hongfu Zhang, Yufeng Huang, Liang Xiong, Rongfeng Zhang, Yunlong Xia, Qing K. Wang, Chengqi Xu

**Affiliations:** 1 Department of Clinical Laboratory, Liyuan Hospital of Tongji Medical College, Huazhong University of Science and Technology, Wuhan, PR China; 2 Human Genome Research Center, Cardio-X Institute, College of Life Science and Technology of Huazhong University of Science and Technology, Wuhan, PR China; 3 State Key Laboratory for Molecular and Developmental Biology, Institute of Genetics and Developmental Biology, Chinese Academy of Sciences, Beijing, PR China; 4 Precision Medical Laboratory, Tongji Medical College, Wuhan Children's Hospital (Wuhan Maternal and Child Health Care Hospital), Huazhong University of Science and Technology, Wuhan, PR China; 5 Department of Cardiology, First Affiliated Hospital of Dalian Medical University, Dalian, PR China

**Keywords:** coronary artery disease, genetics, SNP, *NINJ2*, C/EBP beta

## Abstract

Objective: *NINJ2* regulates activation of vascular endothelial cells, and genome-wide association studies showed that variants in *NINJ2* confer risk to stroke. However, whether variants in *NINJ2* are associated with coronary artery disease (CAD) is unknown.

Methods: We genotyped rs34166160 in *NINJ2* in two independent Chinese GeneID populations which included 2,794 CAD cases and 4,131 controls, and performed genetics association studies. Functional studies were also performed to reveal the mechanisms.

Results: Allele rs34166160 significantly confers risk to CAD in the GeneID Hubei population which contained 1,440 CAD cases and 2,660 CAD-free controls (observed *P_-obs_* = 6.39 × 10^−3^ with an odds ratio (OR) was 3.39, adjusted *P_-adj_* = 8.12 × 10^−3^ with an OR of 3.10). The association was replicated in another population, GeneID Shandong population contained 1,354 CAD cases and 1,471 controls (*P_-obs_* = 3.33 × 10^−3^ with an OR of 3.14, *P_-adj_* = 0.01 with an OR of 2.74). After combining the two populations, the association was more significant (*P_-obs_* = 1.57 × 10^−5^ with an OR of 3.58, *P_-adj_* = 3.41 × 10^−4^ with an OR of 2.80). In addition, we found that rs34166160 was associated with the mRNA expression level of *NINJ2* and the flanking region of rs34166160 can directly bind with transcriptional factor CCAAT-box/enhancer-binding protein beta, and the risk A allele has more transcription activity than non-risk C allele with or without LPS in HUVEC cells.

Conclusions: Our study demonstrates that the functional rare variant rs34166160 in *NINJ2* confers risk to CAD for the first time, and these findings further expand the range of the pathology of CAD and atherosclerosis.

## INTRODUCTION

Atherosclerosis is the leading pathological cause of coronary heart disease (CAD) and ischemic stroke, which claims about 14 million of lives every year and is the main cause of mortality and morbidity worldwide [1, 2]. In China, CAD and stroke are also one of the most common public health problems, and account for 40% of all deaths every year.

Atherosclerosis is a complex trait caused by environmental factors, genetic factors, and their interactions [3]. Risk factors such as abnormal lipid concentrations, obesity, diabetes, smoking, hypertension, physical inactivity, psychosocial situations, and alcohol intake are shared among CAD and ischemic stroke [4, 5]. In addition, epidemiological data shows that genetic background plays a critical role in both CAD and ischemic stroke [6]. Genetic analysis in families or population, such as genome-wide association studies (GWAS) revealed a lot of susceptibility genes/loci confer risk to the incidence of CAD or ischemic stroke, and some of them, including *ANRIL* in 9p21, *PCSK9* in 1p32 and *BRG1* in 19p13 were found to confer risk for both CAD and ischemic stroke [7]. Identification of novel genetic risk variants that are shared by both ischemic stroke and CAD may identify the underlying pathophysiology of atherosclerosis, and facilitate diagnosis, which may ultimately lead to prevention and treatment of ischemic stroke and CAD.

In a previous study, we found that ninjurin2 (encode by *NINJ2*) is expressed in human vascular endothelial cells, and can regulate the expression of genes associated with inflammation and atherosclerosis (IL-1β, TNF-α, IL-8, IL-6, ICAM-1 and E-selectin) in HUVECs. Moreover, we found ninjurin2 can regulate LPS-induced endothelial activation, and the adhesion of monocytes to endothelial cells through the TLR4/ NF-κB/c-jun pathway [8], which proposed that ninjurin2 is a novel regulator of endothelial activation, and may play important roles in the initiation or development of atherosclerosis. Endothelial activation is a crucial step in the initiation and development of the atherosclerosis process. Under irritant stimuli, including dyslipidemia, hypertension, and pro-inflammatory agents, vascular endothelial cells were activated and support monocyte migration into the sub-endothelial space, and then differentiate into macrophages and take up modified lipoproteins to become lipid-laden foam cells. After activation, the normal functions of the arterial endothelium are adversely affected, and this leads to atherosclerosis and cardiovascular diseases, such as CAD and ischemic stroke.

Previously, GWAS and candidate gene based association studies have shown that variants in the *NINJ2* gene confer risk to the incidence of ischemic stroke in several independent populations, including Chinese, Korean, Iranian and Caucasian ancestry [9–14], however, the detailed mechanisms of how *NINJ2* regulates endothelial activation are not very clear, and the relationship between *NINJ2* and endothelial activation also need to be confirmed in other type of atherosclerosis related disease including CAD.

Here, we tested the hypothesis that the association between rs34166160 in *NINJ2* and CAD, which is a low-frequency variant associated with ischemic stroke in Caucasians [15]. We identified rs34166160 in *NINJ2* as a novel genetic risk factor of CAD in the Chinese Han population. Expression quantitative trait loci (eQTLs) were identified between rs34166160 and *NINJ2* mRNA expression in Blood cells. Our further functional studies also demonstrated that the risk A allele of rs34166160 has more transcription activity than the non-risk C allele using the Electrophoretic Mobility Shift Assay (EMSA) and Luciferase Reporter Assay, and may cooperate with transcriptional factor C/EBP beta under the LPS induced endothelial activation. Our data identified rs34166160 in *NINJ2* as a susceptibility locus for CAD, and uncovered a potential mechanism of rs34166160 conferring risk to CAD via regulating the expression of *NINJ2* by binding with C/EBP beta.

## RESULTS

### Characteristics of study subjects

The samples involved in the current research were selected from the GeneID cohort [16–23]. To avoid geographical confounding, our genetic association studies between polymorphisms and disease were carried out in two independent populations. The GeneID-Central population, which contained 1,440 cases with CAD and 2,660 CAD-free controls, was collected from central China (Hubei province). The GeneID-North population consists of 1, 354 CAD patients and 1, 471 CAD-free controls, and are selected from the patients in hospitals in the northern area of China.

Basic clinical and demographic characteristics of the study populations were shown in Table 1. Initially, the association analysis was conducted in the GeneID-Central population that included 1,440 CAD cases and 2,660 non-CAD controls as a discovery population. The mean ages for cases and controls were 60.42 ± 12.14 and 61.25 ± 9.83 years, respectively in the GeneID-Central population. The proportions of females in cases and controls was 38.75% and 38.38%, respectively (Table 1). Association from the discovery population was then verified in another independent replication case-control population (GeneID-North population) and included 1,354 cases and 1,471 controls. The GeneID-North population included 44.60% females in cases and 46.20% in controls. The mean age for the cases was 63.67 ± 12.69 years versus 62.17 ± 10.13 years for controls (Table 1).

**Table 1:** Basic demographic and clinical characteristics of CAD patients and controls involved in the present study.

**Characteristic**	**GeneID-Central Population**		**GeneID-North Population**	
	**Cases (*n* = 1,440)**	**Controls (*n* = 2,660)**	**Cases (*n* = 1,354)**	**Controls (*n* = 1,471)**
Age^a^, mean ± SD, years	60.42 ± 12.14	61.25 ± 9.83	63.67 ± 12.69	62.17 ± 10.13
Sex, Female, *n* (No. %)	558 (38.75%)	1,021 (38.38%)	604 (44.60%)	678 (46.09%)
Hypertension^b^, *n* (No. %)	861 (59.80%)	1,566 (58.87%)	827 (61.08%)^***^	765 (52.0%)
Diabetes^c^, *n* (No. %)	211 (14.65%)^*^	327 (12.30%)	225 (16.62%)^**^	191 (12.99%)
Total Cholesterol , mean ± SD, mmol/L	4.39 ± 1.07^*^	4.31 ± 0.85	4.45 ± 1.11^*^	4.37 ± 1.02
Triglyceride, mean ± SD, mmol/L	1.55 ± 1.12^*^	1.44 ± 1.03	1.48 ± 1.29^*^	1.40 ± 1.33
HDL-C, mean ± SD, mmol/L	1.18 ± 0.44	1.22 ± 0.38	1.10 ± 0.38	1.17 ± 0.45
LDL-C, mean ± SD, mmol/L	2.71 ± 0.80^**^	2.51 ± 0.83	2.64 ± 0.85^*^	2.55 ± 0.66

Data are shown as mean +/− standard deviation (SD) for quantitative variables and percent (%) for qualitative variables. ^*^P < 0.05, ^**^P < 0.01 and ^***^P < 0.001 between cases and controls for quantitative variables and percent (%) for qualitative variables. ^a^Age at the first diagnosis of the disease in CAD case and at enrollment of CAD controls. ^b^Hypertension was defined as a systolic blood pressure of ≧140 mmHg or a diastolic blood pressure of ≧90 mmHg. ^c^Diabetes was defined as ongoing therapy of diabetes or a fasting plasma glucose level of ≥ 7.0 mmol/L.

### Variant rs34166160 in *NINJ2* associates with CAD in two independent populations

The genotype of rs34166160 did not deviate from the Hardy-Weinberg equilibrium in controls in both the discovery GeneID-Central population (*p* = 0.99) and the replication GeneID-North population (*p* = 0.99). The allelic frequencies of rs34166160 in CAD cases were significantly different from those in controls in the discovery GeneID-Central population (Table 2). The frequency of minor A allele of rs34166160 was 0.44% in CAD cases and 0.13% in controls. A significant association was identified with an unadjusted *p* value (*p_-obs_*) of 6.39 × 10^−3^ and an OR of 3.39. The association was also significant (an adjusted *p* or *p_-adj_* = 8.12 × 10^−3^ with and OR of 3.10) after adjusting for covariates (diabetes mellitus, age, hypertension, gender, and plasma lipid concentrations) (Table 2).

**Table 2:** Analysis of allelic association of SNP rs34166160 with CAD in the Chinese Han population.

**Populations (*n*, case/control)**	**Risk Allele**	**Frequency (case/control)**	**Without Adjustment^a^**		**With Adjustment^b^**	
			***P-obs***	**OR (95% CI)**	***P-adj***	**OR (95% CI)**
GeneID-Central Population (1,440/2,660)	A	0.0045 vs.0.0013	6.39 × 10^−3^	3.39 (1.33–8.63)	8.12 × 10^−3^	3.10 (1.30–8.41)
GeneID-North population (1,354/1,471)	A	0.0085 vs. 0.0027	3.33 × 10^−3^	3.14 (1.40–7.04)	0.01	2.74 (1.28–7.66)
Combined population (2,794/4,131)	A	0.0065 vs. 0.0018	1.57 × 10^−5^	3.58 (1.96–6.57)	3.41 × 10^−4^	2.80 (1.30–6.01)

Abbreviations: *P-obs: P* value observed; *P-adj: P* value with adjustment; *OR:* odds ratio. ^a^Unadjusted *P* value and odds ratio (OR) using Chi-square tests with Pearson’s 2 × 2. ^b^Adjusted *P* value by multivariate logistic regression analysis for potential confounders including age, gender, hypertension, diabetes mellitus and lipid concentrations (Tch, TG, HDL-c and LDL-c).

To further validate the association between rs34166160 and CAD, we genotype for the rs34166160 variant in the replication GeneID-North population that contained 1,354 CAD cases and 1,471 controls. Interestingly, we also found that the A allele of the rs34166160 variant confers risk to CAD (*p_-obs_* = 3.33 × 10^−3^ with an OR of 3.14; *p_-adj_* = 0.01 with an OR of 2.74) in the replication population (Table 2).

After performing a meta-analysis, two populations were combined to yield one large population that included 2,794 cases and 4,131 controls. In the combined population, SNP rs34166160 was found to confer a highly significant risk to CAD (*p_-obs_* = 1.57 × 10^−5^, OR = 3.58). Moreover, after adjustment for covariates including sex and age, this association remained significant (*p_-adj_* = 3.41 × 10^−4^, OR = 2.80) in the combined population. These results showed that A allele of rs34166160 in the *NINJ2* gene was significantly associated with CAD in the studied Chinese population.

### Real-time RT-PCR analysis identified that the A allele of rs34166160 is associated with the increased expression level of *NINJ2* mRNA

Rs34166160 (Chr12:623,339, hg38 version of human genome data) is located in the first intron of the *NINJ2* gene. Bioinformatical analysis predicts that rs34166160 is located in an enhancer region according to the Ensembl database (Regulatory Feature: ENSR00000047562, Chr12:621,401-623,800, hg38, http://asia.ensembl.org/), and in a DNase I hypersensitive cluster (chr12:623,241-623,470) in ENCODE data of UCSC genome browser (https://genome.ucsc.edu). ENCODE Candidate Cis-Regulatory Elements (cCREs) also showed that rs34166160 was predicted to be located in an enhancer of *NINJ2* (Chr12:623,197-623,527, ENCODE Accession: EH38E1585530).

**Figure 1:**
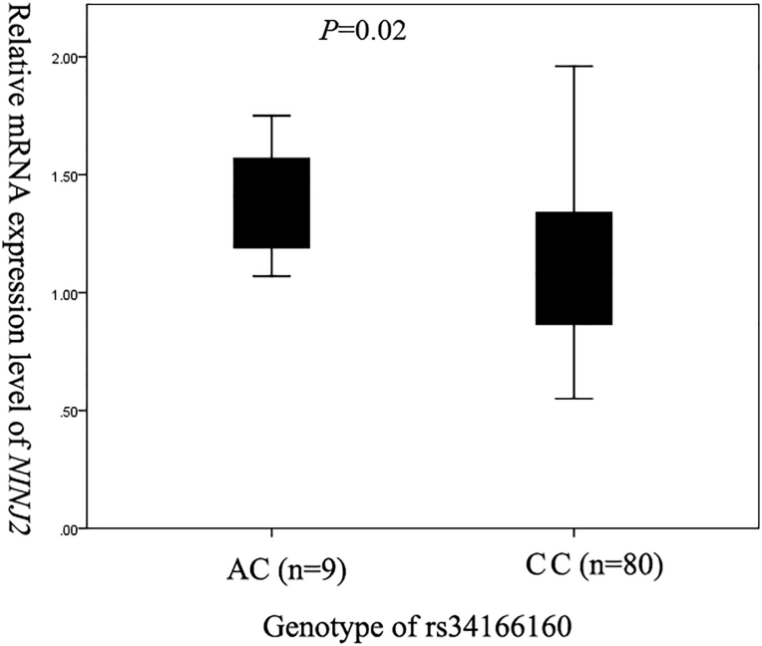
**Assessment of the relationship between *NINJ2* SNP rs34166160 and the expression level of *NINJ2* mRNA by real-time RT-PCR analysis.** Total RNA samples were isolated from blood samples (lymphocytes) of 9 subjects with an AC genotype and 80 subjects with a CC genotype. Real-time PCR analysis was used to analyze the relative expression level of *NINJ2*. A linear regression was used to compare the differences in the mean RQ values between different genotypes (AC and CC) of SNP rs34166160.

Based on its position and the prediction results, we hypothesized that rs34166160 may be associated with the expression level of *NINJ2* and enrolled 89 healthy study subjects undergoing annual physical examinations into GeneID to perform eQTL analysis. We measured the mRNA expression levels of *NINJ2* using blood samples from 9 people with heterozygous genotype of rs34166160 (AC genotype) and 80 people with wildtype of rs34166160 (CC genotype). The results demonstrated that the mean relative expression level of *NINJ2* in the 9 AC genotype carriers was significantly higher than that in the 80 CC genotype carriers (*p* = 0.02, by a Kruskal-Wallis test) (Figure 1). These data suggest that the minor and risk A allele of rs34166160 is associated with an increased expression level of *NINJ2* mRNA.

### Rs34166160 exhibits allelic differences in transcriptional activity via CCAAT-box/enhancer-binding protein beta (C/EBP beta)

**Figure 2:**
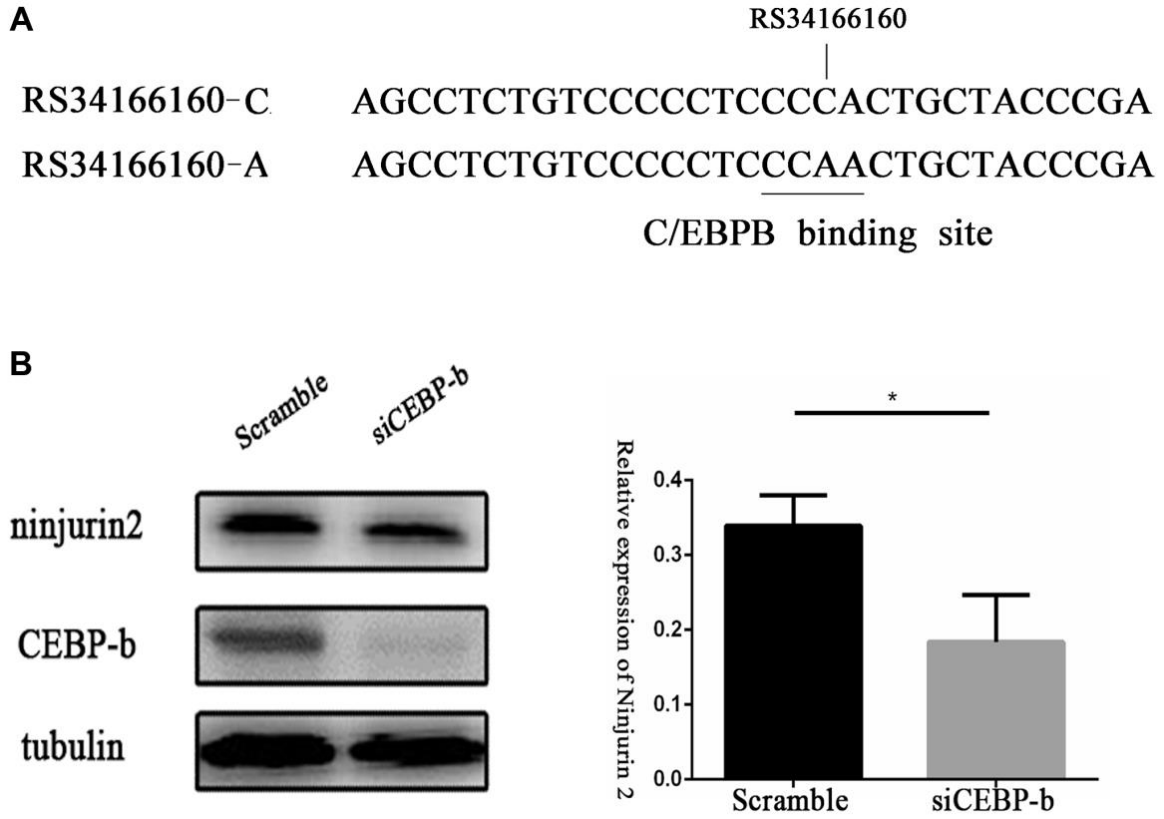
**Transcriptional factor CCAAT-box/enhancer-binding protein beta (C/EBP beta) regulates the expression of *NINJ2*.** (**A**) A schematic diagram shows the predicted binding sites of C/EBP beta in the genomic region overlapping rs34166160 harbored A allele not C allele. (**B**) Knockdown of the expression of C/EBP beta in HUVEC resulted in the decrease of NINJ2 expression (*P* < 0.05). HUVECs were pretreated with scramble siRNA or C/EBP beta siRNA for 48 h, total protein extracts were prepared and blotted with the antibodies specific for *NINJ2*. Three independent experiments were performed. Error bars represent standard deviation (SD).

Through transcript factor binding prediction analysis, we found rs34166160 located in the C/EBP beta binding region in both the PROMO (http://alggen.lsi.upc.es/) and JASPAR (http://jaspar.genereg.net/) database (Figure 2A). First, we tested whether C/EBP beta can affect the expression level of *NINJ2* in Human Umbilical Vein Endothelial Cells (HUVEC). The siRNA approach was used to knockdown the expression of C/EBP beta in HUVEC, and the results showed that compared to controls, expression of *NINJ2* was decreased by knocking down the expression of C/EBP beta in HUVEC (*p* < 0.05) (Figure 2B).

**Figure 3:**
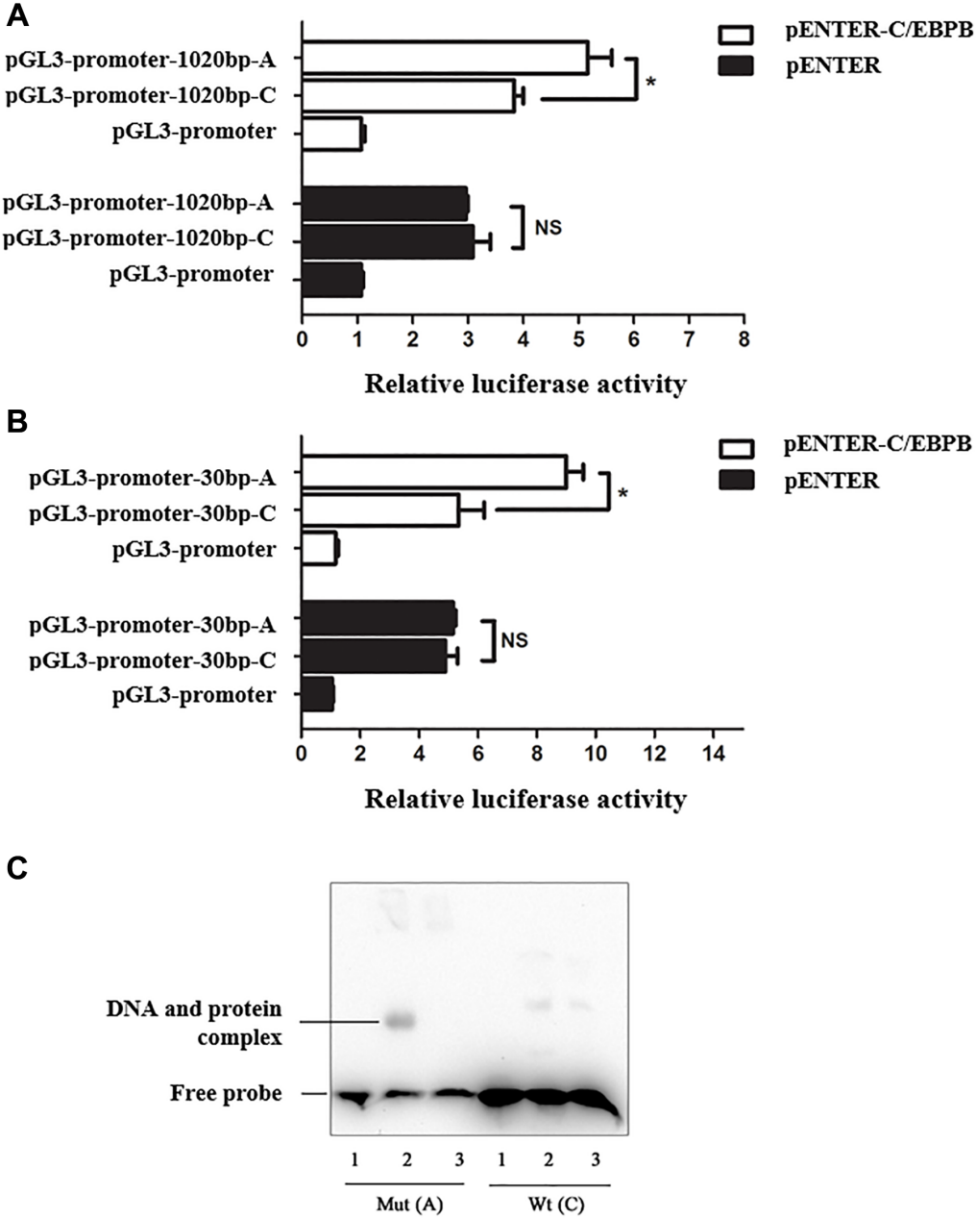
**Risk allele A of rs34166160 exhibits a higher transcriptional activity through interacting with C/EBP beta.** (**A**) Data from luciferase assays in Hela cells. Reporters containing the 1020 bp length genomic fragment overlapping rs34166160 harbored A allele or C allele were co-transfected with pENTER-C/EBP beta or a negative control pENTER into Hela cells. Cells were harvested 48 h after transfection and luciferase activities were measured and normalized to renilla activities. (**B**) Data from luciferase assays in Hela cells. Reporters containing a 30 bp length core genomic segment overlapping rs34166160 harboring the A allele or C allele were co-transfected with pENTER-C/EBP beta or a negative control pENTER into Hela cells. The measurement of luciferase activities was previously described. Three independent experiments were performed. Error bars represent standard deviation (SD). ^*^P < 0.05. (**C**) The risk allele A of rs34166160 can directly bind to the transcription factor C/EBP beta. EMSA probe containing risk allele A or wide type allele C of rs34166160 incubated with (lanes 2–3; lane 5-6) or without (lane 1; lane 4) nuclear extracts from HUVECs transfected with pENTER-C/EBP beta. Lane 1 and lane 4, 5′ -end biotin-labeled probe alone; lane 2 and lane 5, EMSA for 5′ -end biotin-labeled probe and HUVECs nuclear extracts; lane3 and lane 6, excessive unlabelled probe and HUVECs nuclear extract.

To validate whether the genomic region overlapping rs34166160 has regulatory activity and effect as a potential enhancer, we cloned a 1020 bp length genomic fragment overlapping rs34166160 harboring each allele into the PGL-3-promoter luciferase vector which contains a minimal SV-40 promoter. We first performed Dual-luciferase assays in Hela cells which did not express C/EBP beta. The results seemed to be no significant difference in transcriptional activity between fragments containing the C allele and A allele of rs34166160 without C/EBP beta expression in Hela cells (Figure 3A). However, when the luciferase transcriptional reporter assays were performed under the condition of exogenous overexpression of C/EBP beta in Hela cells, the DNA segment containing A allele of rs34166160 was about 1.3-fold than the DNA segment contained C allele of rs34166160 (*p* < 0.05) (Figure 3A). To further investigate the allelic differences in transcriptional activity of rs34166160, we cloned a 30 bp length core genomic segment overlapping rs34166160 harboring each allele into the PGL-3-promoter luciferase vectors containing a minimal SV-40 promoter and performed the transcriptional reporter assays same as above. The data showed the same results as the 1020 bp length segment, and the A allele of rs34166160 showed more luciferase activity than the DNA segment containing the C allele of rs34166160 (*P* < 0.05) (Figure 3B). The data suggest that variant rs34166160 impacts as an enhancer, and thereby may be a functional variant at the 12p13 locus for CAD.

We also performed EMSA assay using HUVEC nuclear extracts and probes flanking the genomic region of rs34166160 which is located in chr12:622,485-622,524 (hg38). Incubation of biotin-labeled probes with different allele of rs34166160 will combined with protein of nuclear extracts from HUVEC, and the probe with A allele showed more binding activity than C allele (Figure 3C). An addition of a 200-fold excess of unlabelled sequence was used to see whether the DNA-protein interaction is specific. The results demonstrated that rs34166160 exhibits allelic differences in transcriptional activity, and may be induced via C/EBP beta.

All these data suggest that the A allele of rs34166160, which confers more risk to CAD, have a higher transcriptional activity compared with the non-risk C allele under the condition of overexpression of C/EBP beta.

### Risk allele A of rs34166160 has a higher transcriptional activity under the condition of endothelial activation by LPS stimuli

Previous studies have shown that *NINJ2* can regulate LPS-induced endothelial activation and inflammation by interacting with TLR4, and the expression of ninjurin2 in HUVEC could be induced by LPS [19]. Vascular inflammation is known to be the critical and initial step in atherosclerosis. In addition, C/EBP beta was reported to mediate LPS-induced endothelial activation and inflammation. Therefore, we hypothesized that the enhancer containing rs34166160 might mediate the LPS-induced increase in NINJ2 expression in HUVECs.

**Figure 4:**
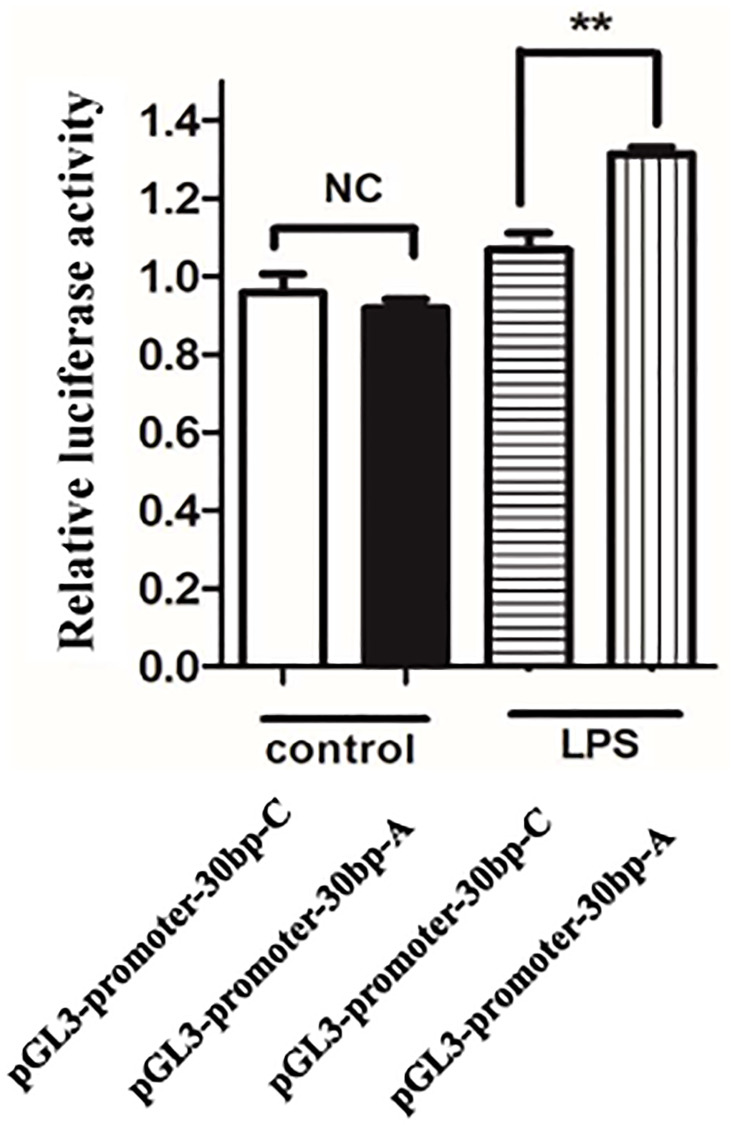
**The risk allele A of rs34166160 has a higher transcriptional activity under the condition of LPS stimuli.** Data from luciferase assays in HUVECs. The PGL-3-promoter luciferase vectors containing the 30 bp length genomic fragment overlapping rs34166160 harboring risk A allele or wide type C allele were transfect into HUVECs for 24 h, and then LPS (1 ug/ml) stimulated for another 24 h. The measurement of luciferase activities was previously described. Three independent experiments were performed. Error bars represent standard deviation (SD). ^*^P < 0.05.

To determine whether rs34166160 is involved in endothelial cell activation and inflammation induced by LPS stimulation, the PGL-3-promoter luciferase vectors containing the 30 bp length genomic fragment overlapping rs34166160 harboring each allele were transfected into HUVEC, and the transcriptional activity was measured using luciferase assay with or without LPS stimuli. The results showed that there were no significant differences in luciferase activity between A allele and C allele without stimulation of LPS (Figure 4). However, under the stimulation of LPS, the A allele of rs34166160 showed more luciferase activity than the DNA segment containing C allele of rs34166160 (Figure 4). These results indicated that the A allele of rs34166160, which is shown to confer more risk to CAD, have a higher transcriptional activity compared with non-risk C allele under inflammatory stimuli, and may have allelic differences in the process of inflammation.

**Figure 5:**
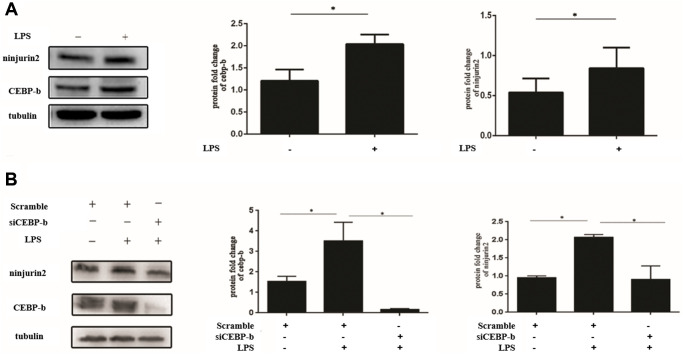
**Increased expression of ninjurin2 induced by LPS stimulation can be blocked by knockdown of C/EBP beta.** (**A**) The expression of C/EBP beta and ninjurin2 in HUVEC in the condition of LPS stimuli. HUVEC cells were plated on 24-well plates for 24 h and followed by stimulation with or without LPS (1 ug/mL) for another 24 h. Total protein extracts were prepared and blotted with the antibodies specific for *NINJ2 or* C/EBP beta. Three independent experiments were performed. Error bars represent standard deviation (SD). (**B**) The increased expression of ninjurin2 induced by LPS stimulation can be blocked by knockdown of C/EBP beta. The si RNA targeted to C/EBP beta were transfect into HUVECs for 24 h, and then LPS (1 ug/ml) stimulated for another 24 h.

We also investigated the expression level of C/EBP beta and ninjurin2 in HUVEC in the condition of LPS stimuli (1 ug/mL), and the results showed that both C/EBP beta and ninjurin2 expression were increased after LPS stimulation (Figure 5A). What is more, the increased expression of ninjurin2 induced by LPS stimulation can be blocked by knockdown of C/EBP beta (Figure 5B). These results indicated that rs34166160 plays an important role in LPS inducing endothelial cell activation through the binding of C/EBP beta transcription factor.

## DISCUSSION

In the current study, we carried out a genetic association study to test the hypothesis that whether rs34166160 in *NINJ2* confers risk to CAD. Our results demonstrated that the minor A allele of a rare variant, rs34166160, in the *NINJ2* gene was conferring risk to CAD in both two independent populations (Table 2). The association of rs34166160 and CAD remained significant after adjustment of the covariates of traditional risk factors (Table 2). Moreover, we found that rs34166160 exhibits allelic differences in transcriptional activity through interacting with C/EBP beta or under stimulation of LPS. As far as we know, this is the first study that showed variant in *NINJ2* associated with the risk of CAD. Considering the previous significant association between rs34166160 and stroke, our study demonstrated that rs34166160 in the *NINJ2* gene may be a shared genetic risk factor for CAD and stroke.

Our association studies between the *NINJ2* variant and CAD were performed in Chinese Han population, and whether the significant association can be replicated in other populations needs to be studied further. We searched the summary statistics of several large GWAS about CAD/MI, including CARDIoGRAMplusC4D Consortium and UK biobank, for the low allele frequency, rs34166160, is not part of the list of the imputation based on meta-GWAS results. Therefore, no data could be found for the association between rs34166160 and related traits. In 2010, we performed a GWAS for CAD in Chinese [19], we also tried to search for the association between rs34166160 and CAD in our previous GWAS. Genotypes of GWAS data were first pre-phased at chromosome levels by SHAPEIT20 and the untyped genotypes were imputed using a merged reference panel from 1000 Genomes Phase 3 data across all 2,504 samples as reference haplotype data by IMPUTE2 with 4 MB per chunk. The other parameters were default parameters in IMPUTE2. The results showed extremely low imputation quality of rs34166160 (Info = 0.04). So, we speculate that it is difficult to impute the genotype of rs34166160 according to previously GWAS data.

The expression of *NINJ2* can be detected in cells directly involved in the inflammatory process of atherosclerosis, including vascular endothelial cells, monocytes, and macrophages [8, 24, 25]. *NINJ2* was also found to be expressed on the cell surface and mediate cell adhesion, which is the critical step of inflammation [8]. Our studies showed that the mechanism of rs34166160 confers risk to CAD and stroke may contribute to the allelic difference of transcriptional activity under the condition of LPS stimuli or C/EBP beta overexpression. Considering that LPS stimuli can cause inflammation [26, 27], and C/EBP beta is one of the most important transcription factors in the inflammation process of atherosclerosis [28, 29], our study provides evidence that *NINJ2* is involved in the pathology of atherosclerosis, and the risk allele A of rs34166160 has a higher sensitivity than C allele in condition of inflammatory stimuli.

Our previous study demonstrated that ninjurin2 can interact with toll-like receptor 4, and activate the inflammation and atherosclerosis pathways through AP-1, c-jun and NF-κB [8]. In the current study, we found that a functional rare variant in *NINJ2* which is located in a C/EBP beta binding site increases the risk of CAD. *C/EBP beta* is a transcription factor belonging to the basic leucine zipper family, and was found to be expressed in multiple types of cells, including myelomonocytic cells, vascular endothelial cells, macrophages and adipocytes [30, 31]. C/EBP beta plays important roles in inflammation and regulates the expression of a panel of genes such as TNF, IL-8, IL-1β, IL-12, IL-6, NF-κB and JNK [31–34]. C/EBP beta also exerts key roles in lipid metabolism and inflammation in both the liver and adipose tissue [35]. Rahman et al. found that the reconstitution of bone marrow from C/EBP β^−/−^ mice to irradiated ApoE^−/−^ mice can result in a decrease in the atherosclerotic lesion size [36]. These studies demonstrated that *C/EBP beta* plays important roles in the process of inflammation and atherosclerosis, and one of the possible mechanisms of *C/EBP beta* affects inflammation and atherosclerosis may contribute to *NINJ2*.

In the current study, we used a LPS-stimulated endothelial cell activation or inflammation model to mimic the initiation of atherosclerosis. Several studies have suggested that infectious agents may initiate or promote the inflammatory process in atherosclerosis. In particular, it is believed that LPS from bacteria such as *Chlamydia pneumonia* [37–39], *Helicobacter pylori* [40, 41], *Porphyromonas gingivalis* [42–44] or gut microbiota may be triggering the secretion of inflammatory cytokines that leads to the recruitment of monocytes/macrophages to the lesions in the process of atherosclerosis or coronary artery disease . Elevated levels of LPS seem to present a risk factor for the development of atherosclerosis [45], whereas injection of LPS has been shown to accelerate formation of atherosclerotic lesions [46]. What is more, LPS binds with TLR4, and can induce the activation of TLR4-MyD88-NF-κB signaling, followed by the release of atherosclerosis related inflammatory factors such as TNF-α, IL-1β, IL-6, and MCP-1, resulting in the development and progression of atherosclerosis in mice models fed with lipids [47, 48]. Therefore, the current study also suggests that *NINJ2* may participate in the process of endothelial cell activation or inflammation stimulated by infectious agents.

In the Chinese population, the minor allele frequency of rs34166160 is extremely low (0.13–0.27%). If the power analysis assumes that the OR for SNP rs34166160 in CAD would be identical to the previously reported HR of 1.8 in stroke, then a limitation of this study may be that the statistical power of our genetic analysis may be insufficient in the two independent populations. Therefore, we cannot exclude the possibility that the significant association between rs34166160 and CAD may represent a false positive result due to the sample size.

In conclusion, for the first time we found that SNP rs34166160 in the *NINJ2* gene is associated with the risk of CAD. We also demonstrated rs34166160 associated with the mRNA expression level of *NINJ2*. In addition, we found that the flanking region of rs34166160 can bind with the transcriptional factor *C/EBP beta* and the A allele have more transcription activity than the C allele. What is more, we found that the A allele has more transcription activity than the C allele under stimulation of LPS. Our results suggest that *NINJ2* is a susceptibility gene for CAD for the first time and that the minor allele A of SNP rs34166160 increases the risk of CAD by altering the binding activity of transcriptional factor *C/EBP beta*.

## MATERIALS AND METHODS

### Study subjects

The samples involved in the current research were selected from the GeneID cohort [17, 19, 21]. All participants are of Han nationality according to their self-description. All the research about human subjects was approved by the ethics committee of the university (Huazhong University of Science and Technology, HUST). The study confirms the guidelines set forth by the Declaration of Helsinki, and all study subjects give written informed consent.

Diagnosis of CAD and myocardial infarction (MI) was carried out by two independent expert cardiologists based on the results of coronary angiography. The criteria of myocardial infarction (MI) and CAD were according to the standard ACC/AHA guidelines [49], and as previous studies [50–53]. Patients with greater than 70% of luminal stenosis in at least one main vessel by coronary angiography, coronary artery bypass graft, percutaneous coronary intervention, and/or MI were diagnosed as CAD patients. The diagnosis of MI was based on typical chest pain of ≥30 min duration, characteristic electrocardiographical patterns of acute MI, and significant elevation of cardiac enzymes (such as CK-MB, lactate dehydrogenase) and troponin I or T. Patients that were identified by angiography with myocardial spasm and myocardial bridge were excluded from the study. Subjects with congenital heart disease, childhood hypertension, and type I diabetes mellitus were also excluded. Control subjects were general population controls and did not show any evidence of CAD or MI as indicated by their medical records or by electrocardiographic analysis.

Basic data such as gender, age, diabetes mellitus, hypertension and lipid concentrations (total cholesterol, triglyceride, LDL cholesterol and HDL cholesterol) were collected from medical records.

### SNP genotyping

Genotyping for SNP rs34166160 was carried out using the TaqMan allelic discrimination assay as previously described [54]. For each round of genotyping, 10 ng of genomic DNA was used in a total volume of 5 μl and containing 1× TaqMan Genotyping Master Mix (Life Technologies) and 1× TaqMan SNP genotyping probe (Life Technologies, CA, USA). The PCR reaction and post-PCR endpoint plate reading was carried out using the Applied Biosystems 7900HT Fast Real-Time PCR System (Life Technologies, CA, USA) according to the manufacturer’s instructions. To ensure the quality of the experiment, appropriate negative controls were included in each round of genotyping. The genotyping results of TaqMan assay were verified by direct Sanger DNA sequencing of 48 subjects randomly selected.

### Real-time quantitative reverse transcription PCR analysis

Real-Time Quantitative Reverse Transcription PCR (qRT-PCR) was used to determine the mRNA expression level of the *NINJ2* among subjects with different genotypes of rs34166160. RNA was obtained from blood sample by Trizol (Life Technologies, CA, USA). Real-Time Quantitative Reverse Transcription PCR Analysis was used to determine the mRNA expression level of the *NINJ2* gene among subjects with different genotypes of rs34166160. Quantitative real-time PCR analysis was carried out according to the MIQE guidelines. Total RNA samples were extracted from human peripheral blood leukocytes using Trizol reagent (Life Technologies, CA, USA). Quantification of RNA samples was performed using a spectrophotometer (NanoDrop, Thermo Scientific, NH, USA). 2 μg of total RNA was used for reverse transcription with Superscript II reverse transcriptase (Life Technologies, CA, USA) and oligo (dT)_18_. A standard two steps real-time PCR assay was performed using an ABI 7500fast Genetic Analyzer (Life Technologies, CA, USA). The primers for *NINJ2* were 5′-ATGCGGCTGAAGGCGGTGCTG-3′ (forward) and 5′-TGGCTGCGTTGTTGAGCTGGTTG-3′ (reverse). The primers for the β-actin reference gene (GeneBank ID: BC014861) are 5′-GGACTTCGAGCAGGAGATGG-3′ (forward) and 5′-GCACCGTGTTGGCGTAGAGG-3′ (reverse). Each PCR reaction was performed in a final volume of 10 μL reaction mixture containing 5 μL of 2X PCR master mixture with ROX (Roche Applied Science, IN), 2 μL of cDNA, 0.4 μL of 10 pM primers, and 2.6 μL of ddH2O. Each reaction was performed in triplicate. The cycling conditions were 95°C for 10 minutes and 40 cycles of 95°C for 15 seconds and 60°C elongation for 45 seconds. After the PCR reaction, Cq values (threshold cycle) of a target gene (*NINJ2*) (Cq T) or reference gene *β-actin* (GeneBank ID: BC014861, Cq E) were computed using the RQ Manager program (version 1.3) and SDS (version 2.3). Reaction with a Cq of ≥40 or with the difference between Cq and mean Cq greater than 0.5 were excluded for further analysis. For each individual, the relative expression level ΔCq (Cq T-Cq E) of a target gene was normalized with the reference gene and then transformed into relative quantity using RQ formula (RQ = 2^−ΔΔCq^, ΔΔCq= individual’s ΔCq-calibrator’s ΔCq). The calibrator was a mixed cDNA sample pooled from 10 randomly selected individuals. The RQ for the calibrator was normalized to 1. After outliers were excluded, a Kruskal-Wallis test (nonparametric analysis of variance) was used to compare the differences for mean RQ values of *NINJ2* between different genotypes of SNP rs34166160.

### Cell culture and treatment

HUVEC were obtained from Wuhan Pricells (Wuhan, Hubei, China). Primary Umbilical Vein Endothelial Cells (HUVECs) were obtained from Wuhan Pricells (Wuhan, Hubei, China). The research was performed using 3 distinct cell batches. HUVECs were maintained in endothelial cell medium (ECM, ScienCell, CA, USA) containing Endothelial Cell Growth Supplement (ECGS, ScienCell, CA, USA), 20% FBS (Gibco, MD, USA), 100 mg/L heparin (Invitrogen, CA, USA), 1 mM Sodium Pyruvate and 1% penicillin/streptomycin (Invitrogen, CA, USA) and used at passage 3–6. Hela cells were cultured in DMEM (Gibco, MD, USA) containing 10% FBS, L-glutamine (2 mM), penicillin G (100 units/ml), streptomycin (10 mg/ml). All cells were grown in an incubator with a humidified atmosphere of 5% CO_2_ at 37°C. Cell were plated in 24-well plates for 24 h and accompanied by incubation with or without LPS (1 ug/mL) for another 24 h.

### Expression vector construction

The predicted 1020 bp length *C/EBP beta* binding region flanking rs34166160 in the first intron of *NINJ2* (Chr12:622,453-623,472, hg38) was generated by PCR and using human genomic DNA as a template. The primer sequences used for amplification are 5′-GAGCTCGGGCTACCTAAAGAGAGGAAGA-3′ contained a *Sac I* restriction enzyme cutting site and 5′-CTCGAGCTTCCCTGATTTTGGCTGGTAC-3′ contained a *Xho I* site. The 1020 bp length PCR products were digested with *Sac I* and *Xho I*, and sub-cloned into the pGL3-Promoter luciferase plasmid (Promega, Madison, WI, USA), resulting in the pGL3-promoter-1020 bp-A or pGL3-promoter-1020 bp-C plasmid. The 30 bp length fragments flanking rs34166160 was synthesized by Genewiz ltd (Suzhou, China). The DNA fragment (5′-CGGGGTACCTCTGTCCCCCTCCCCCACTGCTACCCGAGCCTCGAGCGG-3′ for C allele of rs34166160 and 5′-CGGGGTACCTCTGTCCCCCTCCCCAACTGCTACCCGAGCCTCGAGCGG-3′ for A allele of rs34166160) and their corresponding complement chains were annealed and then sub-cloned into the pGL3-Promoter luciferase plasmid after digesting by *Kpn I* and *Xho I*. Human *C/EBP beta* ORF (NM_005194) was purchased from Origene (Rockville, MD, USA) and sub-cloned into pENTR vector (Thermo fisher, Pittsburgh, PA, USA).

### Luciferase reporter assay

Hela cells co-transfected with 150 ng of either the pENTER control vector or pENTER- *C/EBP beta* and 50 ng pGL3-promoter luciferase reporter plasmids contained C allele or A allele of rs34166160 in a 96-well plate [55]. The transfection efficiency was normalized by co-transfecting of 4 ng pRL-TK plasmid. Cells were lysed using a passive lysis buffer after 48 hours of transfection (Promega, Madison, WI, USA). The dual-luciferase assay was analyzed using the dual-luciferase assay kit (Promega, Madison, WI, USA) using GloMax illuminometer (Promega, Madison, WI, USA).

### Western blot

Western blot was performed as standard protocol [56]. The antibodies used for western blot analysis include an anti- C/EBP beta antibody (1:1500, Proteintech, Wuhan, China), an anti-alpha tubulin (1:5000, Merck Millipore, Darmstadt, Germany) and a goat polyclonal anti-NINJ2 antibody (1:1200, R&D Systems, Minneapolis, MN, USA).

### Electrophoretic Mobility Shift Assay (EMSA)

pENTER- *C/EBP beta* plasmid was transfected into HUVEC for 40 hours using Viafect™ reagent. EMSA probes were designed according to the predicted *C/EBP beta* binding site on SNP rs34166160. The sequence of wild type C allele probe (5′-AGCCTCTGTCCCCCTCCCCACTGCTACCCGA-3′) and the minor A allele probe (5′-AGCCTCTGTCCCCCTCCCAACTGCTACCCGA-3′) was synthesized in Sangong Biotech and with a 5′-end-labeled biotin. NE-PER Nuclear and Cytoplasmic Extract kit was used to extract nuclear proteins (Thermo fisher, Pittsburgh, PA, USA). 2 μg of extracted nuclear protein was hybridized with WT and Mutation DNA probes, respectively. Protein-DNA complexes were analyzed on a 6% nondenaturing PAGE gel. Excessive unlabeled probes were used as competition for EMSA experiments. LightShift™ Chemiluminescent EMSA Kit was used for the EMSA assay (Thermo fisher, Pittsburgh, PA, USA).

### Statistical analysis

For genetic association studies, statistical analysis was carried out as previously reported [17, 21]. Pearson’s 2 × 2 contingency tables and Chi-square tests as implemented in PLINK version 1.08 were used to analyze the SNP allelic association study [57]. Risk factors including lipid concentrations, age, gender, hypertension and diabetes mellitus and lipids profiles were used to adjust the association using multivariate logistic regression analysis. PS:Power and Simple Size Calculation software was used to analyze the statistical power [58]. For western blot and other functional studies, results from at least three independent experiments are shown and presented as mean ± SD. Student’s *t* tests were used for data analysis between the two groups using GraphPad Prism 6 software. *P* < 0.05 means that there is a significant difference between the data and is statistically significant (^*^means *P* < 0.05).
